# Through the Cultural Prism: The Influence of a Distinctive Chinese Personality—Junzi Personality—On Psychosocial Adjustment in China

**DOI:** 10.1002/ijop.70231

**Published:** 2026-06-08

**Authors:** Boqiang Zhao, Changlin Liu, Palizhati Muhetaer, Ping Hu

**Affiliations:** ^1^ Department of Psychology Renmin University of China Beijing China; ^2^ School of Psychology, Hainan Normal University Haikou China

**Keywords:** Chinese culture, cultural confidence, Junzi personality, psychosocial adjustment, self‐control

## Abstract

Grounded in a Chinese indigenous cultural perspective, this study examines how Junzi personality—a culturally grounded personality construct rooted in Confucian philosophy—relates to the psychosocial adjustment of Chinese individuals. It further examines the mediating role of self‐control and the moderating role of cultural confidence. Three substudies collected data from 2259 Chinese participants. Study 1 used longitudinal data (*n* = 205) to examine the effects of Junzi personality on psychosocial adjustment as both a state and a process. Study 2 employed cross‐sectional data (*n* = 1589) to examine whether the associations among Junzi personality, self‐control and psychosocial adjustment were consistent with the proposed pathway. Study 3 used longitudinal data (*n* = 465) to validate the overall model and examine the moderating effect of cultural confidence. The results show that Junzi personality is associated with better psychosocial adjustment; longitudinal evidence further supports the mediating role of self‐control and the moderating role of cultural confidence. These findings highlight Junzi personality as a culturally grounded resource for psychosocial adjustment and clarify a multilevel pathway from culture to individual adjustment. By integrating contemporary psychological theories with Eastern Confucian thought, this study offers an indigenous cultural perspective for future research on personality and adjustment.

## 1 Introduction

Adjustment (or adaptation) is inextricably linked to culture. To adapt to their environments, groups develop shared beliefs, values and practices that constitute their culture (Triandis 1993). However, much existing research has been grounded in Western cultural, philosophical and sociological traditions, which may not fully capture adjustment in non‐Western societies (Chen et al. 2018; Yang 2023). To address this limitation, the present study adopts a Chinese indigenous cultural perspective to examine how sociocultural factors shape Chinese individuals' adjustment to contemporary Chinese society.

Because culture is difficult to capture directly at the societal level, researchers have often examined culturally shaped individual‐level constructs, such as thinking styles and values (Triandis 1993). Personality provides another important example, as personality characteristics and their meanings are closely intertwined with the practices and value systems of particular sociocultural contexts (Zhao and Zhang 2025). For instance, indigenous Chinese personality constructs often emphasise interpersonal relationships and modesty, reflecting traditional Chinese cultural values (Cheung et al. 1996). Therefore, culturally specific personality constructs can serve as prisms through which researchers examine how indigenous cultures shape people's psychology and behaviour. Following this approach, the present study focuses on Junzi personality, an indigenous personality construct derived from Chinese culture.

### 1.1 Junzi Personality

Compared with Western philosophical traditions that emphasise abstract ontology, Confucian philosophy places strong emphasis on moral self‐cultivation and becoming an exemplary person, or Junzi (Liang 2018). Confucius described the qualities, beliefs and values of Junzi and encouraged individuals to cultivate virtue. Under the influence of this cultural ideal, Junzi has long served as an important moral and personality ideal in Chinese society, shaping people's understanding of desirable personal qualities across historical and contemporary contexts (de Bary 2009). Through varying degrees of internalisation of this ideal, individuals may develop Junzi personality to different extents. Therefore, Junzi personality can be viewed as an individual‐level psychological structure that embodies Chinese indigenous culture (Zhao and Zhang 2025).

Ge and Hou (2021) defined Junzi personality as an ideal personality construct rooted in Confucian thought. They operationalised this construct by developing items based on descriptions of Junzi personality in *The Analects*. After testing reliability and validity, they developed a 30‐item questionnaire encompassing five dimensions: (A) ‘wisdom, benevolence, and courage’, (B) ‘respectfulness and propriety’, (C) ‘conversancy with righteousness and cherished rule’, (D) ‘rational behaviour’, and (E) ‘self‐cultivation over contention’. Ge et al. (2021) found that Junzi personality remains applicable to contemporary Chinese individuals and is associated with existing personality constructs. For instance, individuals higher in Junzi personality report higher extraversion, agreeableness, conscientiousness, and openness and lower neuroticism (Ge and Hou 2021).

It is important to clarify that Junzi personality is not treated in the present study as a single elementary trait or as a simple additional dimension to Western trait models. Rather, it is conceptualised as a multidimensional yet coherent higher‐order indigenous personality construct (Zhao and Zhang 2025). This construct is trait‐like because it reflects relatively stable individual differences in cognition and behaviour, while also being culturally embedded because these tendencies are organised around Confucian ideals of virtue, self‐cultivation and interpersonal harmony (Cheung et al. 1996). Thus, although Junzi personality overlaps with broad personality traits such as agreeableness and conscientiousness, it cannot be reduced to these traits. Its distinctiveness lies not in adding isolated behavioural tendencies to existing Western models, but in capturing a culturally meaningful configuration of virtues, values and behavioural dispositions.

### 1.2 Junzi Personality and Psychosocial Adjustment

Psychosocial adjustment represents a critical challenge, as individuals' physical and social environments are constantly changing (Chen et al. 2018; Lawes et al. 2023). Psychologists deconstruct adjustment from two perspectives: state and process. From a state perspective, adjustment refers to the harmony and equilibrium individuals manifest at a given time and is often assessed through mental health, well‐being and social adjustment (Chen et al. 2018; Ellis and Del Giudice 2019). From a process perspective, adjustment involves psychological changes in response to major developmental transitions, such as education, employment or marriage and can be indexed by changes in psychological conditions before and after such events (Chen et al. 2018; Lawes et al. 2023). While most studies have focused on adjustment states, few have examined adjustment processes. Therefore, the present study aims to examine psychological adjustment mechanisms across both states and processes.

Classical Chinese philosophy and contemporary theoretical research suggest that Junzi personality may affect adjustment. Chinese and Confucian cultures contain many ideas that encourage individuals to adjust to their social environment. Specifically, Confucius suggested that Junzi could build appropriate interpersonal relationships while constantly cultivating themselves, adjusting to environmental changes and re‐establishing harmony with the world, thereby reflecting both adjustment states and adjustment processes (de Bary 2009; Watson 2007). From a personality perspective, Hsu's (1972) Psychosocial Homeostasis Theory suggests that the typical Chinese personality emphasises harmony and maintaining good relationships within the social environment. From a cultural perspective, the Theory of Person–Environment Fit proposes that congruence between internal cultural constructs and the surrounding sociocultural context is crucial for successful adaptation (Triandis 1993). As a core expression of Confucian and broader Chinese cultural values, Junzi personality may therefore facilitate adaptation in contemporary Chinese society. Empirical findings support this view, showing that individuals higher in Junzi personality report better interpersonal relationships, more prosocial behaviour, and greater life satisfaction (Ge et al. 2021; Ge and Hou 2021; Zhao and Zhang 2025). However, because some studies suggest that certain indigenous Chinese cultural values do not always translate into adaptive outcomes in modern contexts (Iwamoto et al. 2012), the present study seeks to clarify how Junzi personality shapes both adjustment states and adjustment processes.

### 1.3 The Mediating Role of Self‐Control

Self‐control refers to the ability to regulate thoughts, feelings and actions to resist immediate temptations and impulses in order to achieve long‐term goals (Inzlicht et al. 2021). According to previous theories, self‐control may play a crucial role in linking Junzi personality to psychosocial adjustment. First, self‐control helps individuals adjust to their environments. According to Cybernetic Control Theory, maintaining adaptive states is a long‐term goal, with self‐control acting as a core mechanism to achieve these goals (Inzlicht et al. 2021). Furthermore, the formation and development of self‐control are influenced by sociocultural factors. Self‐Determination Theory (Ryan and Deci 2008) suggests that autonomous motivation promotes the development of positive abilities such as self‐control. Moreover, the development of autonomous motivation is shaped by sociocultural contexts. Finally, Self‐Determination Theory of Vitality (Ryan and Deci 2008) further suggests that cultural contexts shape autonomous motivation for self‐control, thereby helping individuals pursue their goals and adapt to their environments.

In Confucian culture, a similar pathway exists, whereby self‐control serves as a key mechanism through which Junzi personality promotes adjustment. On the one hand, many ancient Confucian scholars argue that Junzi must restrain personal desires to pursue long‐term goals. In Confucian thought, self‐control is a central means of such self‐cultivation (Watson 2007). On the other hand, in Confucian culture, self‐control is an effective method for achieving adjustment or adaptation. According to Confucian philosophy, the presence of ‘ren’ (a noble moral trait) in an individual's life indicates a harmonious and unified adjustment with others and with all things in nature (Liang 2018). So, how does one acquire ‘ren’? Confucius proposed that ‘self‐restraint and the observance of rites lead to “ren”,’ meaning that Junzi achieves ‘ren’ by exercising self‐restraint, thereby attaining adjustment. However, the central role of self‐control in the relationship between Junzi personality and adjustment has mostly been discussed theoretically, with limited empirical evidence. The present study uses cross‐sectional and longitudinal data to examine whether self‐control is involved in the association between Junzi personality and psychosocial adjustment. Specifically, the cross‐sectional study provides preliminary evidence for the pattern of associations among these variables, whereas the longitudinal study offers a stronger test of the proposed mediating process.

### 1.4 The Moderating Role of Cultural Confidence

Intrinsic mechanisms of adaptation influenced by sociocultural factors may also be modulated by cultural confidence. Cultural confidence encompasses cognitive components such as recognition and identification with the dominant culture, as well as the positive emotions generated on this basis (Zhou and Bi 2023). According to Cultural Intelligence Theory (Earley and Ang 2003), cultural confidence enhances individuals' thorough understanding of their native culture, enabling them to recognise which abilities and behaviours are effective and valuable within that cultural context. Furthermore, for individuals with high cultural confidence, sociocultural factors more strongly influence their psychology and behaviour (Zhou and Bi 2023). Therefore, as a cultural product that embodies indigenous Chinese culture at the individual level, the effects of Junzi personality on an individual's abilities may be moderated by Chinese people's confidence in their indigenous culture. Empirical research supports this theoretical perspective. Empirical research also suggests that cultural confidence is associated with stronger self‐awareness and the development of self‐related functions (Attar‐Schwartz and Khoury‐Kassabri 2016; Bi et al. 2022; Zhou and Bi 2023). Based on existing theory and empirical research, this study hypothesises that cultural confidence plays a positive moderating role in the process by which Junzi personality influences self‐control.

### 1.5 The Present Study

The present study addresses several limitations of prior work. Previous studies on adjustment have predominantly been based on Western cultural perspectives, lacking attention to other cultural viewpoints. Additionally, previous research has mainly focused on psychosocial adjustment as a state and seldom examined the underlying mechanisms through which sociocultural factors promote adjustment. Therefore, this study combines both cross‐sectional and longitudinal designs to explore how Chinese culture, through Junzi personality, influences both the state and process of psychosocial adjustment. Moreover, this study investigates the mechanisms through which sociocultural factors promote adjustment, focusing on self‐control and cultural confidence.

**FIGURE 1:**
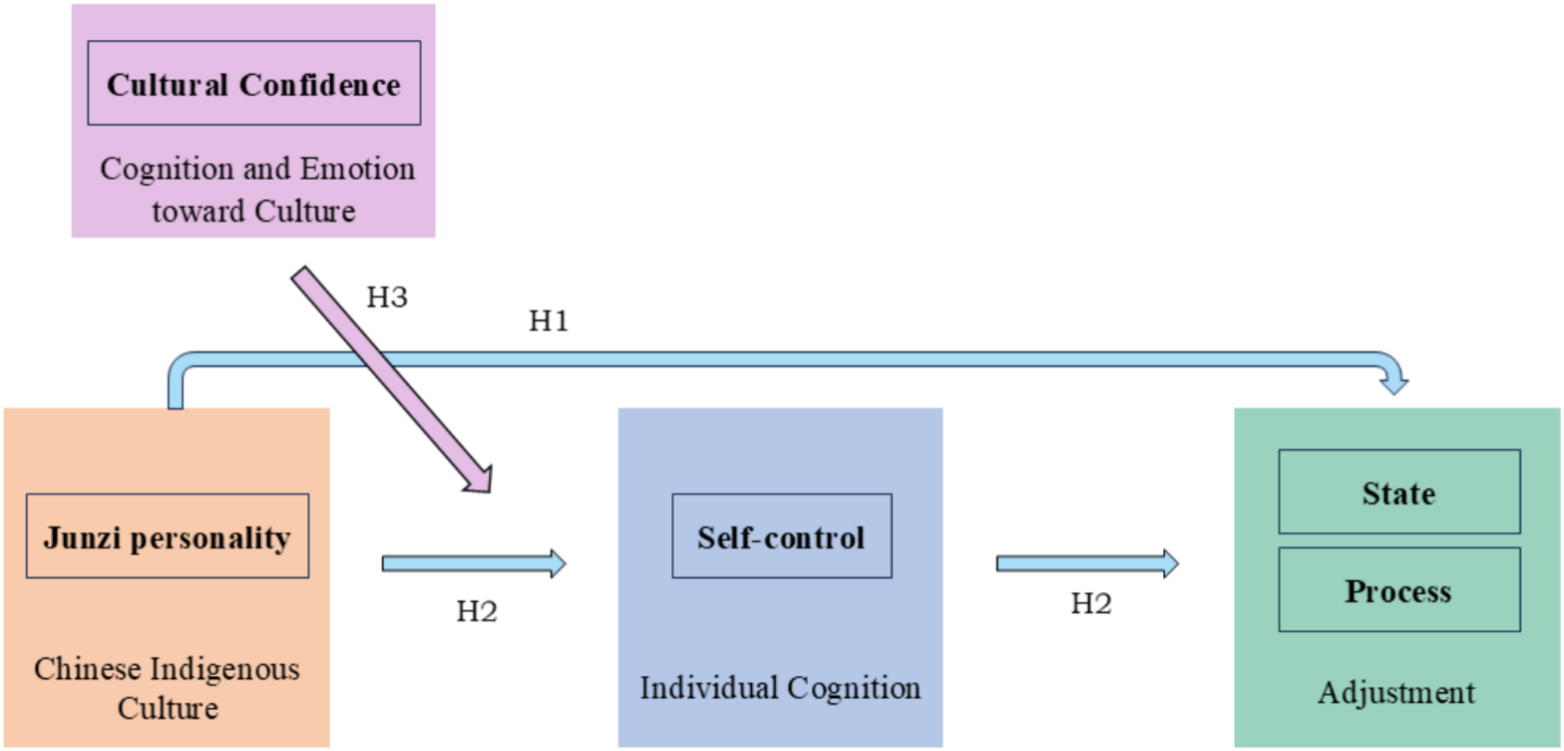
Hypothetical model.

Drawing on Chinese indigenous culture and contemporary psychological theories, the present study hypothesises that Junzi personality promotes both adjustment states and adjustment processes (Hypothesis 1), that self‐control mediates this association (Hypothesis 2) and that cultural confidence moderates the effect of Junzi personality on self‐control (Hypothesis 3). This study comprises three substudies to examine different aspects of the research hypotheses (Figure 1). Study 1 used longitudinal data to examine Hypothesis 1; Study 2 used cross‐sectional data to examine whether the associations among Junzi personality, self‐control and adjustment were consistent with Hypothesis 2 and Study 3 used longitudinal data to provide a stronger test of the mediating process and Hypothesis 3.

## 2 Study 1

Study 1 used well‐established psychological scales to measure variables and examined the differences in the psychological states of adolescents upon entering high school and 3 months later to explore the effect of Junzi personality on the adjustment state and process. Studies 1, 2 and 3 were preregistered before data collection and analysis (https://osf.io/cujph).

### 2.1 Participants and Procedure

Participants were freshmen from a public senior high school in an urban area of Zhejiang Province, China. Prior to testing, informed consent forms were completed by students, parents, teachers and schools. In September 2023 (T1), the first assessment was conducted with 205 students (44.6% male; mean age = 15.36 ± 0.62; mean family economic status = 3.48 ± 1.37, on a 1–7 scale). This time point was chosen as students had been attending the school for nearly a month and were familiar with high school life (Ellis and Del Giudice 2019). The first assessment gathered demographic information, Junzi personality traits and psychological and school adjustments. Three months later, 156 students completed the second assessment (T2), which focused on psychological and school adjustment. To examine whether attrition was systematic, we compared participants who remained in the study with those who dropped out based on T1 demographic variables (gender, age, family economic status) and Junzi personality. Apart from age (*t* = −2.25, *p* = 0.04), no significant differences were found, suggesting that attrition was unlikely to introduce substantial bias. Psychological and school adjustment scores from T1 were used as indicators of adjustment states, and the difference between T2 and T1 scores indicated adjustment processes (Ellis and Del Giudice 2019).

### 2.2 Measures

#### 2.2.1 Junzi Personality

This was measured using the Inventory of Junzi Personality in Confucianism (Ge et al. 2021). The scale consists of 30 items rated on a 7‐point Likert scale (1 = *strongly disagree*; 7 = *strongly agree*), with higher scores indicating a higher level of Junzi personality. Sample items included ‘I consistently uphold a heart of benevolence and virtue’, and ‘I always harmoniously coexist with others’. The Cronbach's *α* for the scale was 0.91 in this study. The CFA results indicated that *χ* ^2^/df = 2.61, CFI = 0.94 and RMSEA = 0.04. The scale has been validated among Chinese adolescents (Ge and Hou 2021).

#### 2.2.2 Psychological Adjustment

This was measured using the Depression–Anxiety–Stress Scale (Lovibond and Lovibond 1995). The scale contains 21 items rated on a 4‐point Likert scale (0 = *never*; 3 = *always*) to assess the three dimensions of negative psychological states: depression, anxiety and stress. Sample items included ‘I felt sad and depressed’, ‘I felt faint’, and ‘I found it difficult to relax’. Higher scores indicated higher levels of depression, anxiety and stress. The Cronbach's *α* was 0.85 (T1) and 0.86 (T2) for anxiety; they were 0.86 (T1) and 0.85 (T2) for stress; they were 0.87 (T1) and 0.86 (T2) for depression. The CFA result at T1 found that *χ* ^2^/df = 3.16, CFI = 0.95 and RMSEA = 0.06. The scale has been validated among Chinese adolescents (Zhao and Hu 2024).

#### 2.2.3 School Adjustment

This was measured using the Scale of School Adjustment (Hou 2016). The questionnaire contained 27 items using a 5‐point Likert scale (1 = *not at all*, 5 = *very much*), with higher scores indicating higher school adjustment. Sample items included ‘I am very content with my school life’. The Cronbach's *α* was 0.92 and 0.89 for T1 and T2, respectively. The CFA result at T1 found that *χ* ^2^/df = 3.24, CFI = 0.89 and RMSEA = 0.10.

### 2.3 Results and Discussion

#### 2.3.1 Testing of Common Method Bias

Using the Harman single‐factor test, the results indicated 30 factors with eigenvalues greater than one. The largest eigenvalue was 37.88, which explained 24.76% of the variance.

#### 2.3.2 Descriptive Statistics and Correlations

Table 1 presents the descriptive statistics and correlations. At T1, students' psychological adjustment was at a moderate level and their school adjustment was at a relatively good level. By T2, both psychological adjustment and school adjustment had slightly improved, although none of these changes reached statistical significance (anxiety: *t* = −0.74, *p* = 0.46; stress: *t* = −1.22, *p* = 0.23; depression: *t* = −0.99, *p* = 0.33; school adjustment: *t* = 1.63, *p* = 0.19). Correlation analyses indicate significant correlations among the main variables.

**TABLE 1:** Descriptive statistics and correlations in Study 1 (*n* = 205).

	*M* (SD)	1	2	3	4	5	6	7	8	
1. T1 Junzi personality	5.20 (0.65)	—								
2. T1 anxiety	1.63 (0.50)	−0.22***	—							
3. T1 stress	1.72 (0.57)	−0.25***	0.81***	—						
4. T1 depression	1.36 (0.47)	−0.26***	0.61***	0.72***	—					
5. T1 school adjustment	4.09 (0.51)	−0.43***	−0.56***	−0.54***	−0.49***	—				
6. T2 − T1 anxiety	−0.03 (0.42)	−0.04	0.37***	0.19**	0.01	−0.09	—			
7. T2 − T1 stress	−0.05 (0.47)	−0.05	0.25***	0.43***	0.18*	−0.08	0.54***	—		
8. T2 − T1 depression	−0.04 (0.40)	−0.15*	0.11	0.17**	0.25**	−0.10*	0.52***	0.60***	—	
9. T2 − T1 school adjustment	0.05 (0.34)	0.13*	−0.10	−0.07	−0.01	0.23***	−0.10	−0.11	−0.19**	—

*Note*: T1, Time 1; T2, Time 2; T2 − T1, difference between outcome variables at Time 1 and Time 2.

* *p* < 0.05.

** *p* < 0.01.

*** *p* < 0.001.

#### 2.3.3 Junzi Personality and Psychosocial Adjustment State

After controlling for age, gender and family economic status, T1 Junzi personality significantly negatively predicted T1 anxiety (*β* = −0.21, *p* < 0.001, 95% CI = [−0.34, −0.09], ∆*R* ^2^ = 0.048), T1 stress (*β* = −0.25, *p* < 0.001, 95% CI = [−0.37, −0.12], ∆*R* ^2^ = 0.063) and T1 depression (*β* = −0.25, *p* < 0.001, 95% CI = [−0.38, −0.13], ∆*R* ^2^ = 0.057). Conversely, it positively predicted T1 school adjustment (*β* = 0.42, *p* < 0.001, 95% CI = [0.30, 0.53], ∆*R* ^2^ = 0.183).

#### 2.3.4 Junzi Personality and the Psychosocial Adjustment Process

After controlling for age, gender and family economic status, T1 Junzi personality significantly positively predicted T2‐T1 school adjustment (*β* = 0.13, *p* = 0.04, 95% CI = [0.01, 0.26], ∆*R* ^2^ = 0.023).

## 3 Study 2

The results of Study 1 indicated that Junzi personality positively predicted adjustment states and processes. Study 2 aimed to examine whether the associations among Junzi personality, self‐control and psychosocial adjustment were consistent with the proposed theoretical pathway, using cross‐sectional data from a larger and more diverse sample. Because cross‐sectional data cannot establish temporal ordering or causal relations, Study 2 was not intended to provide a definitive test of mediation. Instead, it served as a preliminary large‐sample examination of the correlational pattern underlying the proposed model (Maxwell and Cole 2007).

### 3.1 Participants and Procedure

This study recruited participants through Wenjuanxing (https://www.wjx.cn). Before the measurements, the participants were required to read and sign an informed consent form. Following the removal of responses that were consistently identical or for which the completion time was < 3 min, valid responses from 1589 participants (56.5% female, *M*_age_ = 22.43, SD_age_ = 6.47) were included in the study.

### 3.2 Measures

#### 3.2.1 Junzi Personality

The measurement of Junzi personality was consistent with that of Study 1. The Cronbach's *α* was 0.91. The CFA results indicated that *χ* ^2^/df = 3.17, CFI = 0.91 and RMSEA = 0.07.

#### 3.2.2 Self‐Control

This construct was measured using the Chinese version of the Self‐Control Scale (Tan and Guo 2008), which consists of 19 items rated on a 5‐point scale (1 = *strongly disagree*, 5 = *strongly agree*). Higher scores indicated better self‐control. Example items included ‘I can resist temptation well’. The Cronbach's *α* was 0.88. The CFA results indicated that *χ* ^2^/df = 4.62, CFI = 0.96 and RMSEA = 0.06.

#### 3.2.3 Psychological Adjustment

The measurement of psychological adjustment was consistent with Study 1. The Cronbach's *α* was 0.83 for anxiety; it was 0.90 for stress; it was 0.84 for depression. The CFA results indicated that *χ* ^2^/df = 3.99, CFI = 0.92 and RMSEA = 0.07.

#### 3.2.4 Social Adjustment

This construct was measured using the Social Adjustment Assessment Questionnaire (Liu et al. 2012). The questionnaire consisted of 50 items rated on a 5‐point scale (1 = *not at all*, 5 = *very much*). It is categorised into positive and negative adjustment states. Sample items include ‘I am willing to sincerely help others’ and ‘I am not interested in others and do not want to care about them’. The Cronbach's *α* for positive and negative adjustment were 0.95 and 0.93, respectively. The CFA results indicated that *χ* ^2^/df = 5.34, CFI = 0.88 and RMSEA = 0.09.

### 3.3 Results and Discussion

#### 3.3.1 Testing of Common Method Bias

Harman's single‐factor test indicated that 20 factors had eigenvalues greater than one. The largest eigenvalue was 30.68, accounting for 25.57% of the variance.

#### 3.3.2 Descriptive Statistics and Correlations

Tables S1 presents the descriptive statistics and correlations among the variables. The results indicate significant correlations among the main variables.

#### 3.3.3 Junzi Personality and Psychosocial Adjustment State

After controlling for gender, age and economic status, Junzi personality significantly negatively predicted anxiety (*β* = −0.32, *p* < 0.001, 95% CI = [−0.37, −0.27], ∆*R* ^2^ = 0.109), stress (*β* = −0.32, *p* < 0.001, 95% CI = [−0.37, −0.28], ∆*R* ^2^ = 0.119), depression (*β* = −0.31, *p* < 0.001, 95% CI = [−0.36, −0.27], ∆*R* ^2^ = 0.107) and negative social adjustment (*β* = −0.47, *p* < 0.001, 95% CI = [−0.51, −0.43], ∆*R* ^2^ = 0.237). Meanwhile, it positively predicted positive social adjustment (*β* = 0.66, *p* < 0.001, 95% CI = [0.62, 0.70], ∆*R* ^2^ = 0.436).

#### 3.3.4 Cross‐Sectional Associations Among Junzi Personality, Self‐Control and Adjustment

We examined the associations among Junzi personality, self‐control and psychosocial adjustment indicators. The detailed results are presented in Table 2. Junzi personality was positively associated with self‐control. Self‐control was negatively associated with anxiety, depression, stress and negative social adjustment, and positively associated with positive social adjustment. These results indicate that the pattern of associations was consistent with the proposed theoretical pathway. However, because all variables in Study 2 were measured at the same time point, these findings should be interpreted as preliminary correlational evidence rather than evidence for a causal mediating process.

**TABLE 2:** Exploratory estimates of cross‐sectional indirect associations in Study 2 (*n* = 1589).

	*M*: self‐control		Y1: anxiety		Y2: stress		Y3: depression		Y4: negative social adaptation		Y5: positive social adaptation	
	*β* (SE)	95% CI	*β* (SE)	95% CI	*β* (SE)	95% CI	*β* (SE)	95% CI	*β* (SE)	95% CI	*β* (SE)	95% CI
X: Junzi personality	0.55*** (0.02)	[0.50, 0.60]	−0.08** (0.03)	[−0.13, −0.02]	−0.10* (0.03)	[−0.11, −0.09]	−0.10** (0.03)	[−0.16, −0.03]	−0.21*** (0.03)	[−0.26, −0.14]	0.50*** (0.03)	[0.45, 0.55]
M: Self‐control	—	—	−0.46*** (0.03)	[−0.52, −0.40]	−0.45*** (0.03)	[−0.51, −0.40]	−0.42*** (0.03)	[−0.48, −0.37]	−0.46*** (0.02)	[−0.51, −0.42]	0.27*** (0.02)	[0.22, 0.32]
U1: Gender	−0.80** (0.02)	[−0.12, −0.04]	−0.004 (0.02)	[−0.05, 0.04]	0.03 (0.02)	[−0.01, 0.10]	0.05* (0.02)	[0.01, 0.10]	0.04 (0.02)	[−0.002, 0.08]	0.02 (0.02)	[−0.02, 0.05]
U2: Age	−0.13*** (0.02)	[−0.17, −0.09]	0.06* (0.03)	[0.01, 0.11]	0.10*** (0.02)	[0.05, 0.14]	0.11*** (0.03)	[0.06, 0.16]	0.14*** (0.02)	[0.10, 0.18]	0.03 (0.02)	[−0.01, 0.07]
U3: Economic status	0.02 (0.02)	[−0.02, 0.07]	−0.08** (0.02)	[−0.13, −0.03]	−0.05* (0.02)	[−0.03, −0.01]	−0.13*** (0.02)	[−0.17, −0.08]	−0.08*** (0.02)	[−0.13, −0.05]	0.08*** (0.02)	[0.04, 0.11]
	*R* ^2^ *=* 0.33, *F =* 15.74***		*R* ^2^ *=* 0.29, *F =* 14.85***		*R* ^2^ *=* 0.30, *F =* 14.91***		*R* ^2^ *=* 0.29, *F =* 15.41***		*R* ^2^ *=* 0.44, *F =* 21.32***		*R* ^2^ *=* 0.49, *F =* 26.39***	
Indirect effect	—		−0.25*** (0.02)	[−0.30, −0.22]	−0.25*** (0.02)	[−0.29, −0.21]	−0.23*** (0.02)	[−0.27, −0.20]	−0.25*** (0.02)	[−0.29, −0.22]	0.15*** (0.02)	[0.12, 0.18]

Abbreviations: *β*, standardised parameter estimates; SE, robust standard error; 95% CI = 95% confidence intervals.

* *p* < 0.05.

** *p* < 0.01.

*** *p* < 0.001.

## 4 Study 3

Study 2 showed that the cross‐sectional associations among Junzi personality, self‐control and psychosocial adjustment were consistent with the proposed theoretical pathway. However, because cross‐sectional data cannot establish temporal ordering or causal relationships (Maxwell and Cole 2007), Study 3 used three‐wave longitudinal data to provide a stronger test of the mediating role of self‐control and the moderating role of cultural confidence.

### 4.1 Participants and Procedure

We recruited senior college students who had graduated and secured employment via an online platform. The first assessment (T1, June 2023) collected data from 465 students, including demographics, Junzi personality traits and cultural confidence. The second assessment (T2, September 2023) included 441 participants who had started working, with data on self‐control, affect, life satisfaction and subjective happiness. The third assessment (T3, December 2023) involved 420 participants who had been employed for 3 months, collecting data on affect, life satisfaction and subjective happiness. Changes in these variables between T3 and T2 were used to measure the long‐term adjustment process. The mean age of participants at T1 was 22.90 ± 2.21 years, with 208 of them being male (45.9%). As in Study 1, we assessed whether attrition was systematic by comparing the demographic variables (gender, age and economic status) and Junzi personality of those who remained with those who dropped out. No significant differences were found, suggesting that attrition did not introduce substantial bias in Study 3.

### 4.2 Measures

#### 4.2.1 Junzi Personality

The measurement of Junzi personality was consistent with that of Study 1. The Cronbach's *α* was 0.93. The CFA results indicated that *χ* ^2^/df = 2.58, CFI = 0.96 and RMSEA = 0.08.

#### 4.2.2 Cultural Confidence

This construct was measured using a Cultural Confidence Inventory (Zhou and Bi 2023). The inventory consists of 10 items rated on a 7‐point Likert scale (1 = *strongly disagree*; 7 = *strongly agree*), with higher scores indicating a higher level of Chinese cultural confidence. Sample items included ‘I am generally content with Chinese culture’. The Cronbach's *α* was 0.93. The CFA results indicated that *χ* ^2^/df = 2.94, CFI = 0.93 and RMSEA = 0.09.

#### 4.2.3 Self‐Control

The measurement of self‐control was consistent with Study 2. The Cronbach's *α* was 0.84. The CFA results indicated that *χ* ^2^/df = 2.07, CFI = 0.96 and RMSEA = 0.06.

#### 4.2.4 Subjective Well‐Being

Based on previous research (Lawes et al. 2023), this study used the average difference between life satisfaction and the positive–negative affect difference as an indicator of subjective well‐being. Higher scores on this measure reflect greater subjective well‐being.

Life satisfaction was measured using the Satisfaction with Life Scale (Diener 1984). The scale comprises five items rated on a 7‐point scale (1 = *strongly disagree*, 7 = *strongly agree*), with higher scores indicating greater life satisfaction. Sample items include ‘I am satisfied with my life’. The Cronbach's *α* at the two time points was both 0.87. The CFA result at T2 found that *χ* ^2^/df = 1.94, CFI = 0.97 and RMSEA = 0.04.

Positive–negative affect was measured using the Positive Affect and Negative Affect Schedule (PANAS, Watson et al. 1988). This scale comprised 10 positive and 10 negative emotion words (1 = *strongly disagree*, 5 = *strongly agree*). The Cronbach's *α* at the two time points was 0.79 and 0.87, respectively. The CFA result at T2 found that *χ* ^2^/df = 3.16, CFI = 0.95 and RMSEA = 0.06.

### 4.3 Results and Discussion

#### 4.3.1 Testing of Common Method Bias

Harman's single‐factor test indicated 28 factors with eigenvalues greater than one. The largest eigenvalue was 27.64, accounting for 20.79% of the variance.

#### 4.3.2 Descriptive Statistics and Correlations

The correlations between the variables are listed in Table S2. The results indicate significant correlations among the main variables.

#### 4.3.3 Junzi Personality and Psychosocial Adjustment Process

After controlling for gender, age and economic status, regression analysis revealed that T1 Junzi personality significantly positively predicted the difference in negative affect between T3 and T2 (*β* = 0.13, *p* = 0.04, 95% CI = [0.01, 0.25], ∆*R* ^2^ = 0.015), life satisfaction (*β* = 0.24, *p* < 0.001, 95% CI = [0.10, 0.38], ∆*R* ^2^ = 0.052) and subjective well‐being (*β* = 0.20, *p* = 0.01, 95% CI = [0.05, 0.34], ∆*R* ^2^ = 0.034). Furthermore, T1 Junzi personality exhibited a marginally significant positive predictive effect on the difference in positive affect between T3 and T2 (*β* = 0.13, *p* = 0.06, 95% CI = [−0.01, 0.28], ∆*R* ^2^ = 0.016).

#### 4.3.4 The Mediating Role of Self‐Control and the Moderating Role of Cultural Confidence

We employed the PROCESS Model 8 to test the moderated mediation model. The detailed results are presented in Table 3. The moderated mediation analysis revealed that the interaction term between T1 Junzi personality and T1 cultural confidence significantly predicted T2 self‐control (*β* = 0.30, *p* = 0.003, 95% CI = [0.10, 0.50]). Simple slope analysis indicated that for individuals with lower cultural confidence (M‐1SD), T1 Junzi personality did not significantly predict T2 self‐control (*β* = 0.09, *p* = 0.26, 95% CI = [−0.10, 0.28]); however, for individuals with higher cultural confidence (*M* + 1SD), T1 Junzi personality significantly predicted T2 self‐control (*β* = 0.44, *p* < 0.001, 95% CI = [0.27, 0.61]). These results suggest that T2 self‐control mediates the effects of T1 Junzi personality on T3–T2 life satisfaction and subjective well‐being, and that T1 cultural confidence moderates the influence of T1 Junzi personality on T2 self‐control.

**TABLE 3:** Moderated mediation model in Study 3 (*n* = 465).

	*M*: T2 self‐control		Y1: T3 − T2 positive affect		Y2: T3 − T2 negative affect		Y3: T3 − T2 life satisfaction		Y4: T3 − T2 well‐being	
	*β* (SE)	95% CI	*β* (SE)	95% CI	*β* (SE)	95% CI	*β* (SE)	95% CI	*β* (SE)	95% CI
X: T1 Junzi personality	0.28*** (0.08)	[0.14, 0.42]	0.16*** (0.08)	[0.01, 0.31]	0.16* (0.06)	[0.03, 0.28]	0.21** (0.07)	[0.06, 0.35]	0.16* (0.08)	[0.01, 0.31]
M: T2 Self‐control	—	—	0.01 (0.07)	[−0.14, 0.16]	−0.01 (0.06)	[−0.13, 0.11]	0.17* (0.07)	[0.03, 0.32]	0.16* (0.07)	[0.01, 0.30]
W: T1 Cultural confidence	−0.004 (0.11)	[−0.22, 0.21]	−0.25* (0.11)	[−0.47, −0.02]	−0.26** (0.10)	[−0.44, −0.07]	−0.13 (0.11)	[−0.35, 0.09]	−0.08 (0.11)	[−0.30, 0.14]
X × W	0.30** (0.10)	[0.10, 0.50]	−0.04 (0.11)	[−0.25, 0.17]	0.01 (0.09)	[−0.17, 0.19]	0.03 (0.10)	[−0.18, 0.23]	0.01 (0.11)	[−0.20, 0.22]
U1: Gender	−0.17* (0.07)	[−0.30, −0.04]	0.05 (0.07)	[−0.09, 0.19]	0.34*** (0.06)	[0.22, 0.46]	0.01 (0.07)	[−0.13, 0.14]	−0.12 (0.07)	[−0.26, 0.02]
U2: Age	−0.14* (0.07)	[−0.28, −0.004]	0.03 (0.07)	[−0.12, 0.18]	0.20*** (0.06)	[0.07, 0.32]	0.07 (0.07)	[−0.07, 0.21]	−0.01 (0.07)	[−0.16, 0.13]
U3: Economic status	−0.10 (0.07)	[−0.24, 0.04]	0.10 (0.07)	[−0.05, 0.24]	0.18*** (0.06)	[0.05, 0.30]	0.05 (0.07)	[−0.09, 0.20]	0.00 (0.07)	[−0.14, 0.15]
	*R* ^2^ *=* 0.14, *F =* 5.56***		*R* ^2^ *=* 0.07, *F =* 1.97		*R* ^2^ *=* 0.13, *F =* 3.77***		*R* ^2^ *=* 0.11, *F =* 3.29***		*R* ^2^ *=* 0.07, *F =* 2.26*	

	X → M		X → Y1		X → Y2		X → Y3		X → Y4	
	*β* (SE)	95% CI	*β* (SE)	95% CI	*β* (SE)	95% CI	*β* (SE)	95% CI	*β* (SE)	95% CI
W: *M*‐1SD	0.09 (0.10)	[−0.10, 0.28]	0.18 (0.10)	[−0.01, 0.38]	0.15 (0.08)	[−0.01, 0.32]	0.19 (0.10)	[−0.01, 0.38]	0.15 (0.10)	[−0.04, 0.35]
W: *M*	0.28*** (0.08)	[0.14, 0.42]	0.16 (0.08)	[−0.01, 0.31]	0.16* (0.06)	[0.03, 0.28]	0.21* (0.07)	[0.06, 0.35]	0.16* (0.08)	[0.01, 0.31]
W: *M* + 1SD	0.44*** (0.08)	[0.27, 0.61]	0.13 (0.10)	[−0.06, 0.32]	0.16* (0.08)	[0.00, 0.32]	0.22* (0.10)	[0.03, 0.41]	0.16 (0.10)	[−0.03, 0.35]

Abbreviations: *β*, standardised parameter estimates; SE, robust standard error; 95% CI, 95% confidence intervals.

* *p* < 0.05.

** *p* < 0.01.

*** *p* < 0.001.

## 5 General Discussion

The results across all three studies consistently support Hypothesis 1. Although some research has suggested that traditional cultural beliefs may hinder adjustment in modern society (Iwamoto et al. 2012), our findings indicate that indigenous cultural–psychological structures such as Junzi personality can also facilitate adjustment in contemporary China. This pattern is consistent with the Theory of Person–Environment Fit, which suggests that adaptation is facilitated when individuals' cultural beliefs are aligned with the sociocultural context in which they live (Triandis 1993). Although Chinese society has undergone substantial change, Confucian ideas remain influential in contemporary Chinese culture (Yang 2023). Individuals with higher Junzi personality may therefore exhibit greater congruence with this cultural context and be better equipped to adapt to Chinese society.

These findings may also offer implications for understanding the relationship between culture and adaptation in dynamic contexts. Berry's acculturation framework suggests that adaptation is not simply adjustment to a fixed cultural environment, but involves cultural and psychological changes when individuals and groups encounter changing or plural cultural contexts (Berry 2005). From this perspective, the positive role of Junzi personality suggests that certain indigenous cultural ideals may continue to support adaptation, not by remaining unchanged, but by being internalised as individual‐level psychological structures that remain congruent with the current sociocultural environment (Zhao and Zhang 2025). Junzi personality may thus represent a process through which Confucian ideals are transformed into relatively stable personal dispositions and enacted in ways that reproduce and reinterpret culturally valued patterns of thought and behaviour (Chen 2025). Thus, the present findings are consistent with the possibility that indigenous traditions may be selectively maintained and psychologically reconfigured through personality, thereby continuing to shape adaptation in a changing society.

The results of Studies 2 and 3 were generally consistent with Hypothesis 2, with Study 2 showing the expected cross‐sectional association pattern and Study 3 providing longitudinal evidence for the mediating role of self‐control. Taken together, these findings suggest that self‐control may be an important mechanism linking Junzi personality to psychosocial adjustment. According to Self‐Determination Theory of Vitality (Ryan and Deci 2008), self‐control is an effective means through which people maintain adaptation within their environments, and sociocultural contexts shape the autonomous motivation that underlies self‐control. Confucian philosophy has emphasised that resisting inappropriate impulses and desires is a key route through which Junzi achieves harmony between the self and the surrounding world (Zhao and Zhang 2025). However, few empirical studies have examined the role of self‐control in how culture influences adjustment. By combining cross‐sectional and longitudinal designs, the present study provides empirical support for this culturally embedded pathway and demonstrates a multilevel process in which sociocultural factors enhance psychosocial adjustment by shaping individuals' self‐control.

The results of Study 3 also support Hypothesis 3 and indicate that people who feel more confident in Chinese culture exhibit a stronger association between Junzi personality and self‐control. This finding is broadly consistent with prior research (Attar‐Schwartz and Khoury‐Kassabri 2016). According to Cultural Intelligence Theory, individuals high in cultural confidence are more motivated to understand the meanings that the dominant culture ascribes to specific behaviours and to develop the psychological and behavioural patterns it endorses (Bi et al. 2022). Because self‐control is widely valued in Chinese society (Yang 2023), individuals with stronger confidence in Chinese culture may be more likely to view self‐control as meaningful and to translate Junzi‐related values into self‐control efforts. In this sense, cultural confidence should not be interpreted simply as protecting indigenous culture from external change. Rather, it may help individuals selectively recognise, maintain and enact culturally meaningful values in changing external systems (Zhou and Bi 2023). Whether cultural confidence buffers cultural change, facilitates selective cultural continuity or supports the transformation of indigenous values in new contexts remains an important question for future research.

The present study has several theoretical contributions. Much of psychological theory adopts a universalistic cultural perspective and cannot fully specify how particular indigenous cultures shape psychological tendencies and adjustment (Ge et al. 2021). By treating Junzi personality as a culturally grounded personality construct rather than a mere supplement to Western trait models, the present study shows how personality can be organised around locally meaningful ideals, values and behavioural expectations rather than by adding an isolated element to the existing ‘skeleton’ of personality. Moreover, by identifying self‐control and cultural confidence as mediating and moderating mechanisms, respectively, the study clarifies how culturally shaped cognition and cultural identification link indigenous culture to individual adjustment. In this way, the study extends cultural psychology beyond a predominantly universalistic perspective and clarifies one concrete pathway through which indigenous culture becomes psychologically instantiated. Meanwhile, the results provide empirical support for theories explaining how culture influences psychological functioning and behaviour, including person–environment fit and self‐determination perspectives. The present findings also have practical insights. First, educational and counselling programmes could integrate Confucian ideas of Junzi virtues into interventions that target self‐control during key transitions such as entering high school or the workforce. Second, strengthening cultural confidence may not only increase the likelihood that people endorse Junzi‐related values, but also motivate them to translate these values into concrete self‐control efforts that support their psychosocial adjustment.

Although this study has several contributions, it also has limitations. First, because Junzi personality originates from Confucianism, future research could examine whether it also functions in other East Asian societies influenced by Confucian culture, such as Japan and Korea (Liang 2018). Second, this study was conducted among Chinese participants living in contemporary Chinese society, making it suitable for examining the fit between Junzi personality and the mainstream Chinese cultural context but insufficient for addressing acculturation in culturally plural settings. Future research could examine whether Junzi personality facilitates or constrains adjustment among ethnic minority individuals, Chinese immigrants, international students or bicultural individuals who navigate multiple cultural systems, thereby clarifying whether it mainly supports adaptation to culturally congruent environments or also helps individuals negotiate cultural change and transform their social contexts.

## 6 Conclusions

Adjustment is a critical issue faced by people worldwide. The findings of this study reveal that Junzi personality, rooted in Chinese Confucian culture, enhances individuals' psychosocial adjustment to contemporary Chinese society. It also improves their overall adjustment processes following significant events. Furthermore, self‐control mediates the positive impact of Junzi personality on psychosocial adjustment. Finally, cultural confidence positively moderates the mediating role of self‐control.

## Author Contributions

**Boqiang Zhao:** conceptualization, investigation, methodology, writing – review and editing, writing – original draft, software, data curation, formal analysis, validation. **Changlin Liu:** conceptualization, methodology, writing – original draft, investigation. **Palizhati Muhetaer:** conceptualization, writing – review and editing, writing – original draft. **Ping Hu:** project administration, resources, supervision, writing – review and editing, writing – original draft, funding acquisition, conceptualization, visualization.

## Funding

Project supported by National Social Science Foundation of China (Major Program) (19ZDA021).

## Ethics Statement

This study was approved by the Ethics in Human Research Committee of the local university. All procedures adhered to the ethical guidelines outlined in the 1964 Declaration of Helsinki.

## Consent

All participants read and completed informed consent forms.

## Conflicts of Interest

The authors declare no conflicts of interest.

## Supporting information


**Table S1:** Descriptive statistics and correlations in Study 2 (*n* = 1589).
**Table S2:** Descriptive statistics and correlations in Study 3 (*n* = 465).

## Data Availability

The data that support the findings of this study are available from the corresponding author upon reasonable request.
