# Supplementary material for: Through the Cultural Prism: The Influence of a Distinctive Chinese Personality—Junzi Personality—On Psychosocial Adjustment in China
Source: Int J Psychol. 2026 Jun 8;61(4):e70231. doi: 10.1002/ijop.70231 (PMC13246274; doi:10.1002/ijop.70231)
Supplement: Supplementary file 1 — Table S1: Descriptive statistics and correlations in Study 2 (n = 1589). Table S2: Descriptive statistics and correlations in Study 3 (n = 465). [file IJOP-61-e70231-s001.docx]

**Supplementary Table 1** Descriptive statistics and correlations in Study 2 (*n* = 1589)

|  | *M(SD)* | 1 | 2 | 3 | 4 | 5 | 6 | 7 |
| --- | --- | --- | --- | --- | --- | --- | --- | --- |
| 1. Junzi personality | 4.97(.65) | - |  | - |  |  |  |  |
| 2. Self-control | 3.08(.61) | .55^***^ | - |  |  |  |  |  |
| 3. Anxiety | 1.73(.50) | -.34^***^ | -.52^***^ | - |  |  |  |  |
| 4. Stress | 1.94(.61) | -.36^***^ | -.53^***^ | .77^***^ | - |  | - |  |
| 5. Depression | 1.46(.52) | -.34^***^ | -.50^***^ | .75^***^ | .77^***^ | - |  |  |
| 6. Negative social adjustment | 2.13(.57) | -.50^***^ | -.62^***^ | .65^***^ | .69^***^ | .70^***^ | - |  |
| 7. Positive social adjustment | 3.51(.63) | .66^***^ | .54^***^ | -.43^***^ | -.45^***^ | -.44^***^ | -.64^***^ | - |

*Note.* **p* < .05, ***p* < .01, ****p* < .001.

**Supplementary Table 2** Descriptive statistics and correlations in Study 3 (*n* = 465)

|  | *M(SD)* | 1 | 2 | 3 | 4 | 5 | 6 | 7 |
| --- | --- | --- | --- | --- | --- | --- | --- | --- |
| 1. T1 Junzi personality | 4.87(.79) | - |  | - |  |  |  |  |
| 2. T1 Cultural confidence | 5.41(.64) | .14^*^ | - |  |  |  |  |  |
| 3. T2 Self-control | 3.11(.62) | .21^**^ | .02 | - |  |  |  |  |
| 4. T3-T2 Positive affect | .57(.71) | .16^*^ | -.14 | .02 | - |  |  |  |
| 5. T3-T2 Negative affect | .31(1.00) | .26^***^ | -.16^*^ | -.07 | .21^**^ | - |  |  |
| 6. T3-T2 Life satisfaction | -.19(1.11) | .25^***^ | -.06 | .20^**^ | .27^***^ | .12^***^ | - |  |
| 7. T3-T2 Well-being | -.03(.62) | .16^*^ | -.02 | .21^**^ | .44^***^ | -.24^***^ | .90^***^ | - |

*Note.* T1 *=* Time 1, T2 *=* Time 2, T3 *=* Time 3, T3-T2 *=* Difference between outcome variables at Time 3 and Time 2. **p* < .05, ***p* < .01, ****p* < .001.
